# Forefoot deformation during the stance phase of normal gait

**DOI:** 10.1186/1757-1146-5-S1-P12

**Published:** 2012-04-10

**Authors:** Saartje Duerinck, Friso Hagman, Ilse Jonkers, Peter Vaes, Peter Van Roy

**Affiliations:** 1 Department of Experimental Anatomy, Vrije Universiteit Brussel, Brussels, 1090, Belgium; 2 Department of Physical Therapy, Vrije Universiteit Brussel, Brussels, 1090, Belgium; 3 Department of Human Biomechanics & Biometrics, Vrije Universiteit Brussel, Brussels, 1090, Belgium; 4 Department of Biomedical Kinesiology, Katholieke Universiteit Leuven Belgium, Leuven, 3000, Belgium

## Background

During human walking the ankle-foot complex executes seemingly contradictory functions: (1) stabilization of the human body at initial contact, (2) shock absorption during early stance [1-3], (3) Storing elastic energy during midstance and (4) providing a strong lever for push of during final stance [1]. This quadrupled function inevitably demands a transfer from a flexible and compliant foot towards a rigid lever [1]. Despite the viable role of the forefoot in this transfer, knowledge concerning the deformation of the forefoot is limited. The aim of this study is to provide a more detailed description of deformation occurring at the level of the forefoot during the stance phase of normal human walking.

## Materials and methods

Using a seven-camera motion capture system (250Hz), a pressure platform (500Hz) and a forceplate (1250Hz), we measured forefoot deformation through kinematic and pressure related outcome measures in 60 healthy subjects.

## Results

**Figure 1:**
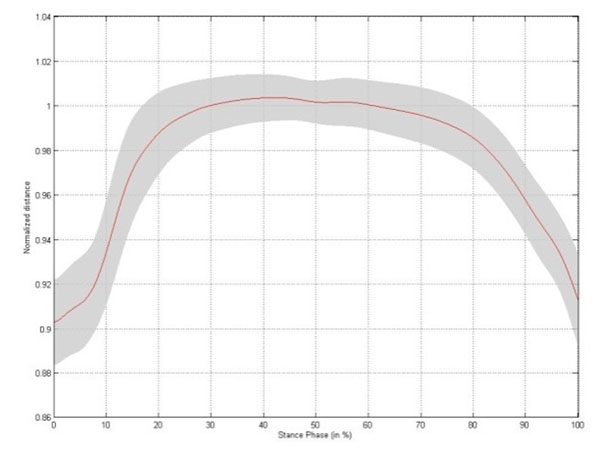
Changes in distance between metatarsal head I - V, I - II/III and II/III – V and in metatarsal arch height: Changes in distance between metatarsal head I and metatarsal head V throughout stance phase for the left foot,

**Figure 2:**
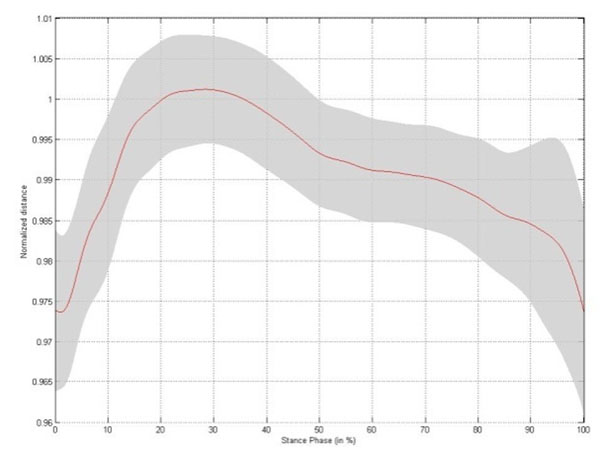
Changes in distance between the base of metatarsal I and the base of metatarsal V throughout stance phase for the left foot,

**Figure 3:**
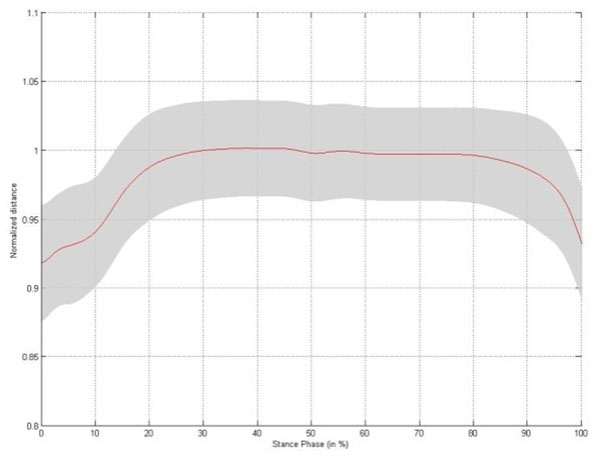
Changes in distance between metatarsal head I and metatarsal head II/III throughout stance phase for the left foot,

**Figure 4:**
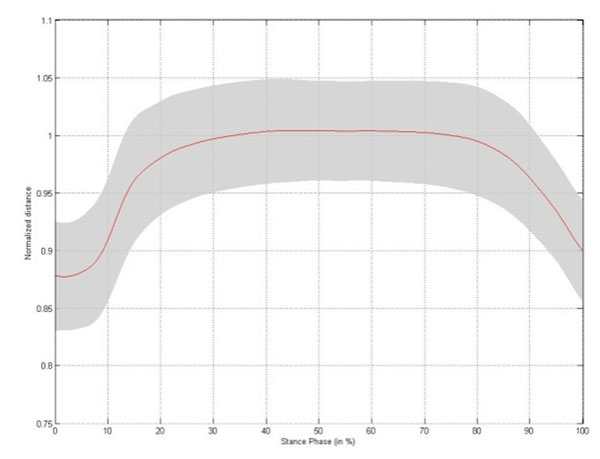
Changes in distance between metatarsal head II/III and metatarsal head V throughout stance phase for the left foot

**Figure 5:**
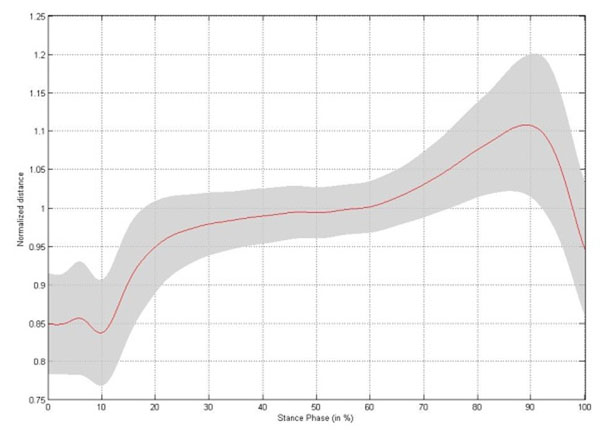
Changes in medio-lateral arch height throughout stance phase for the left foot

Small but significant changes in intermetatarsal distance are established during stance phase, with the largest change occurring between metatarsal head II/III and V (Table 1). The changes in intermetatarsal distance and metatarsal arch height show slightly different patterns. Both patterns are characterized by a rapid increase in distance during initial stance, reaching a stable platform throughout midstance. At the end of stance phase the intermetatarsal distances rapidly decrease to baseline, whereas the metatarsal arch height increases till a maximum at heel off (Figure 1-5).

**Table 1:** Parameters characterizing the changes in medio-lateral arch height and mutual distances between metatarsal head I, II/III and V and metatarsal base I and V during stance phase and for the different subphases

	StPh (mm)	HC (mm)	MF (mm)	MS (mm)	IPO (mm)	FPO (mm)
**Max. MedioLat Height**	1.13 ± 0.08	0.87 ± 0.07	0.87 ± 0.06	1.01 ± 0.04	1.13 ± 0.08	1.05 ± 0.10
**Min. MedioLat Height**	85.95 ± 8.95	4.39 ± 2.50	12.34 ± 3.32	47.25 ± 12.02	87.39 ± 7.73	95.88 ± 1.27
**Max. distance HMTI-HMTV**	1.01 ± 0.01	0.92 ± 0.02	0.96 ± 0.02	1.01 ± 0.01	1.00 ± 0.01	0.94 ± 0.02
**Min. distance HMTI-HMTV**	0.90 ± 0.02	0.90 ± 0.02	0.92 ± 0.02	0.96 ± 0.02	0.94 ± 0.02	0.91 ± 0.02
**Max. distance HMTI-HMTII/III**	1.01 ± 0.04	0.94 ± 0.04	0.95 ± 0.04	1.01 ± 0.03	1.01 ± 0.02	0.97 ± 0.04
**Min. distance HMTI-HMTII/III**	0.91 ± 0.04	0.92 ± 0.04	0.93 ± 0.04	0.95 ± 0.04	0.97 ± 0.04	0.93 ± 0.04
**Max. distance HMTII/III- HMTV**	1.01 ± 0.04	0.89 ± 0.05	0.94 ± 0.05	1.01 ± 0.04	1.01 ± 0.04	0.93 ± 0.04
**Min. distance HMTII/III- HMTV**	0.87 ± 0.05	0.87 ± 0.05	0.89 ± 0.05	0.94 ± 0.48	0.93 ± 0.04	0.90 ± 0.04
**Max. distance BMTI-BMTV**	1.00 ± 0.01	0.99 ± 0.01	0.99 ± 0.01	1.00 ± 0.01	1.00 ± 0.01	1.00 ± 0.01
**Min. distance BMTI-BMTV**	0.97 ±0.01	0.97 ± 0.01	0.99 ± 0.01	0.99 ± 0.01	0.98 ± 0.01	0.97 ± 0.01

Legend: StPh = stance phase, HC = heel contact, MF = metatarsal forming, MS = midstance, IPO = initial propulsion, FPO = final propulsion, max. = maximum, min. = minimum, HMT = head metatarsal, BMT = base metatarsal

High correlation values (>0.7 or <-0.7) are found between temporal pressure and temporal kinematic parameters.

## Conclusion

Through stance the forefoot deforms according to a specific pattern, which is predominantly determined through forefoot-ground interaction. In addition, the changes in forefoot kinematics in combination with temporal contact data argue the existence of a mediolateral metatarsal arch and suggest the existence of an inverse arch during metatarsal forming and final propulsion phase.

## References

[B1] Jenkyn TR Anas K Nichol A Foot segment kinematics during normal walking using a multisegment model of the foot and ankle complex J Biomech Eng 2009 131 034504 10.1115/1.2907750 19154075

[B2] Winter DA Energy generation and absorption at the ankle and knee during fast, natural, and slow cadences Clin Orthop Relat Res 1983 131 147 154 6839580

[B3] Ren LHD Ren LQ Nester C Tian LM a phase-dependent hypothesis for locomotor functions of human foot complex J Bionic Eng 2008 5 175 180 10.1016/S1672-6529(08)60022-0

